# A Multiaxial Fatigue Life Prediction Approach Accounting for Additional Strengthening Effect Based on Energy-Critical Plane Model

**DOI:** 10.3390/ma18174089

**Published:** 2025-09-01

**Authors:** Bo Wang, Jianxiong Gao, Yiping Yuan, Jianxing Zhou, Qin Cheng, Rui Pan

**Affiliations:** 1 School of Mechanical Engineering, Xinjiang University, Urumqi 830017, China; wb15101366871@163.com (B.W.); yipingyuan@163.com (Y.Y.); xju_zhjx@xju.edu.cn (J.Z.); qazedc686@163.com (R.P.); 2 Xinjiang Intelligent Equipment Research Institute, Aksu 843000, China; 18290781233@163.com

**Keywords:** multiaxial fatigue, nonproportional additional strengthening, additional damage, critical plane method, life prediction

## Abstract

Accurate estimation of multiaxial fatigue life plays a critical role in maintaining the structural integrity and operational reliability of mechanical components subjected to complex loading conditions. Under non-proportional loading, fatigue life tends to decrease significantly due to the emergence of additional damage mechanisms, such as dislocation accumulation, cyclic hardening, and accelerated propagation of micro-cracks. This study conducts a systematic investigation into the primary factors that influence fatigue behavior under non-proportional loading conditions. A novel damage factor is proposed, which quantifies the additional strengthening effects caused by complex stress and strain interactions. Based on this factor, a new prediction model is developed through the combination of critical plane theory and an energy-based framework. This model captures the influence of non-proportional strengthening on fatigue strength with improved accuracy. Experimental validation is carried out using En8, TC4, and Al7050-T7451 materials under tension and torsion loading conditions. Comparative analysis with three conventional models shows that the proposed method improves the accuracy of predictions and offers a dependable approach for practical engineering applications.

## 1. Introduction

In engineering applications, most mechanical components are subjected to alternating loads, which can be categorized as either uniaxial or multiaxial [1]. Under such cyclic loading conditions, fatigue damage emerges as the dominant failure mode [2,3,4], significantly impacting the reliability and durability of mechanical systems [5]. While uniaxial fatigue analysis is relatively well established, multiaxial fatigue introduces greater complexity due to the simultaneous action of multiple stress components [6], varying loading paths [7], and phase differences between stress and strain [8]. Compared to uniaxial loading, multiaxial loading exerts a more pronounced effect on the fatigue life of mechanical components [9]. This is attributed to factors such as additional hardening effects, microstructural characteristics, material anisotropy, and localized stress concentrations. These factors give rise to complex fatigue damage mechanisms, including non-proportional hardening, stress redistribution, and early crack initiation on various material planes. Consequently, fatigue failure mechanisms under multiaxial loading are significantly more intricate than those under uniaxial conditions, making accurate fatigue life prediction a persistent challenge.

In recent decades, significant efforts have been devoted to developing reliable methods for predicting fatigue life under multiaxial stress states. Despite substantial progress, achieving high prediction accuracy remains a critical challenge. A major difficulty lies in the selection and quantitative characterization of damage coefficients, which are essential for capturing a material’s fatigue response under complex loading. The proper identification and modeling of these coefficients directly influence the reliability and applicability of predictive models. Continued research in this area is crucial for enhancing structural design, optimizing performance, and ensuring the safety and longevity of components in service.

According to the literature, multiaxial fatigue life prediction methods can be broadly classified into three categories: the equivalent stress/strain method [10], the critical plane method [11], and the energy-based approach [12]. Each method has its own advantages and limitations, depending on the loading conditions [13]. Under non-proportional loading, the rotation of the principal stress and strain axes leads to the activation of multiple slip systems, disrupts the stabilization of dislocation arrangements, and results in additional non-proportional hardening [14]. This phenomenon ultimately reduces the fatigue life of engineering components. Since the pioneering systematic non-proportional loading experiments conducted by Lamba and Sidebottom [15], the phenomena of enhanced material response and reduced fatigue life have garnered substantial attention. As a result, fatigue-resistant design strategies that consider non-proportional additional hardening, creep damage, and their interactions have become a key focus of research. To quantify the additional fatigue damage caused by non-proportional loading, researchers have proposed various non-proportionality coefficients. For instance, Fatemi et al. [16] introduced a parameter involving the maximum normal stress on the critical plane to highlight its role in accelerating crack propagation. Xu et al. [17] performed fatigue experiments on 304 stainless steel specimens subjected to proportional and non-proportional loading conditions. They developed a numerical approach to model cyclic deformation and calculate damage accumulation and found that the numerical results closely matched the experimental data with minimal errors. Zhao et al. [18] investigated the fatigue behavior of 7075-T651 aluminum alloy and suggested a modification to the SWT model. Chen et al. [19] extended the SWT model by incorporating shear stress and strain. Lu et al. [20] developed an effective energy model that accounts for uniaxial cyclic plastic work as well as additional hardening effects. Xu et al. [21] combined virtual strain energy with the critical plane approach to establish a fatigue model. Ince et al. [22] presented fatigue damage parameters based on generalized strain energy and generalized strain amplitude. Shang et al. [23] characterized additional fatigue damage based on normal strain fluctuations, emphasizing their influence on crack separation and early failure. Zhao et al. [24] proposed a strain-based non-proportional damage parameter defined on a sub-critical plane, providing a broader understanding of multiaxial damage mechanisms. Gates et al. [25] investigated the interaction between shear and normal stress under multiaxial loading. They introduced a normalization method for shear stress using the maximum normal stress in the FS model, which not only improved prediction accuracy but also clarified the physical interpretation of fatigue failure. A multiaxial fatigue life prediction model was developed by Liu et al. [26] using the equivalent stress field intensity approach. This model considers the impact of the non-uniform stress field near the notch root, including effects such as non-proportional additional hardening and stress gradient. While these models effectively address certain aspects of non-proportional fatigue damage, many still neglect the influences of phase difference, additional material hardening, and microstructural behavior. Since fatigue damage depends not only on stress and strain amplitudes but also on material-specific responses and phase interactions between loading components, a comprehensive model should integrate these factors along with fatigue crack evolution mechanisms.

This study systematically investigates the reduction in fatigue life caused by additional hardening effects under non-proportional multiaxial loading, a crucial factor affecting the performance and reliability of engineering components. The research emphasizes the influence of phase differences between stress components and material hardening parameters on fatigue damage evolution, providing detailed insight into the underlying mechanisms of multiaxial fatigue. To address the challenges in evaluating material hardening behavior, a novel method is proposed to determine the hardening coefficient using a static strength factor, offering a practical and efficient way to characterize material response under cyclic loading. Additionally, fatigue life prediction is enhanced by integrating an energy-based method with the critical plane approach, enabling the model to accurately capture the complex interactions between multiaxial stresses, material hardening, and fatigue damage accumulation. This comprehensive approach highlights the importance of explicitly considering non-proportional hardening effects and provides a robust predictive tool for practical engineering applications.

## 2. Energy-Critical Plane Model

### 2.1. Energy-Critical Plane

In fatigue damage analysis, the energy-based approach assumes that each loading cycle introduces additional energy into mechanical components, resulting in irreversible material degradation [27]. As the number of loading cycles increases, the accumulated strain energy progressively builds up, increasing the likelihood of crack formation in specific directions [28]. When the accumulated fatigue damage reaches a critical threshold, failure occurs. This approach effectively characterizes both deformation and damage evolution under cyclic loading and is widely used in fatigue life prediction. However, because energy is a scalar quantity, it cannot fully capture the directional nature of crack initiation and propagation on its own [29]. Under complex multiaxial loading conditions, crack growth is also governed by the stress state, strain state, and material microstructure, making a purely energy-based assessment insufficient [30].

**Figure 1:**
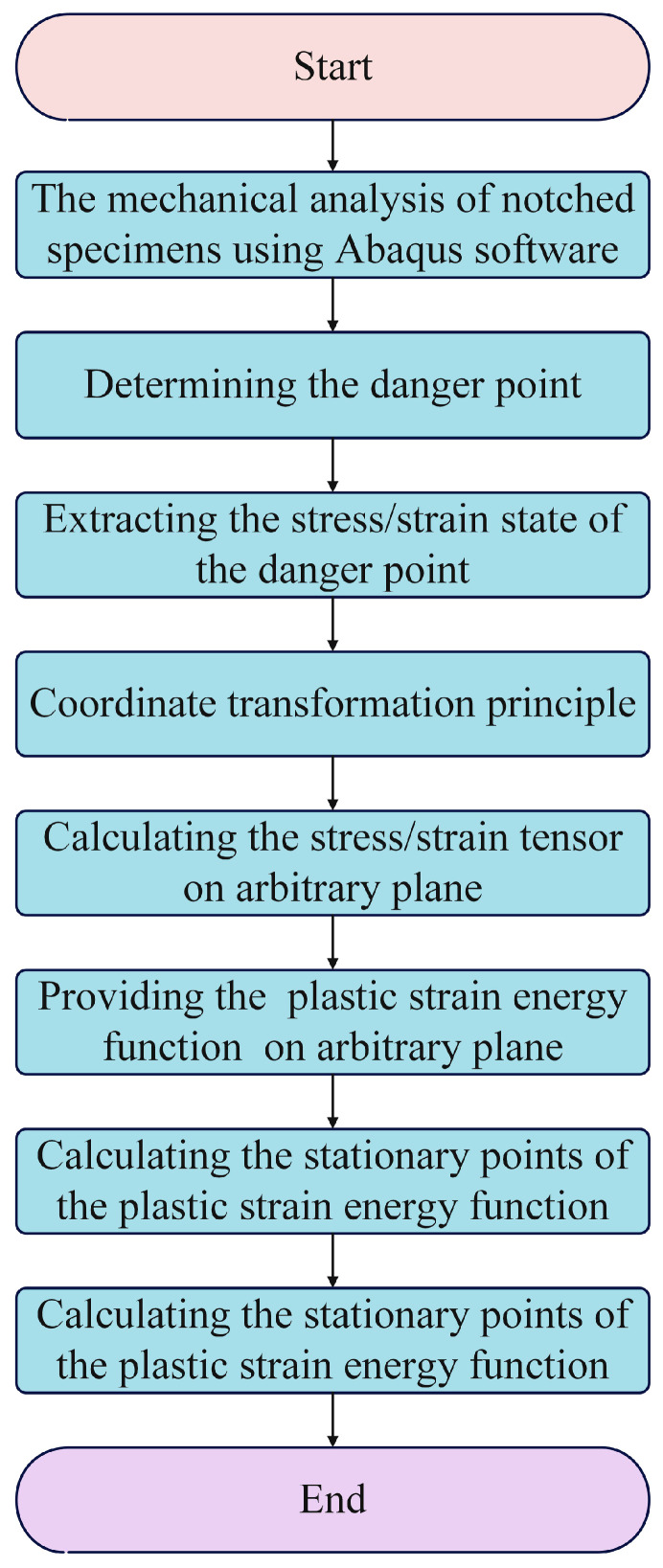
Flowchart for the determination of the energy-critical plane.

To overcome this limitation, the critical plane method is often adopted, as it identifies the fracture plane and provides a physically meaningful description of crack evolution. This method enables accurate prediction of the crack initiation location and improves the overall estimation of fatigue failure. By combining the energy-based approach with the critical plane concept, predictive capability is further enhanced through the integration of energy accumulation and crack orientation analysis. This combination offers a more comprehensive understanding of fatigue damage mechanisms and increases the applicability and reliability of prediction models in engineering practice. Consequently, the energy–critical plane method has become increasingly important in fatigue analysis. Figure 1 illustrates the identification process associated with this method and establishes the relationship between energy accumulation and crack initiation direction for improved life prediction.

### 2.2. Coordinate Transformation Principle

The stress tensor and strain tensor of the elements are given below:

(1) $$\sigma =\left[\begin{array}{ccc} \sigma_{11} & \sigma_{12} & \sigma_{13}\\ \sigma_{21} & \sigma_{22} & \sigma_{23}\\ \sigma_{31} & \sigma_{32} & \sigma_{33} \end{array}\right];\varepsilon =\left[\begin{array}{ccc} \varepsilon_{11} & \varepsilon_{12} & \varepsilon_{13}\\ \varepsilon_{21} & \varepsilon_{22} & \varepsilon_{23}\\ \varepsilon_{31} & \varepsilon_{32} & \varepsilon_{33} \end{array}\right]$$

**Figure 2:**
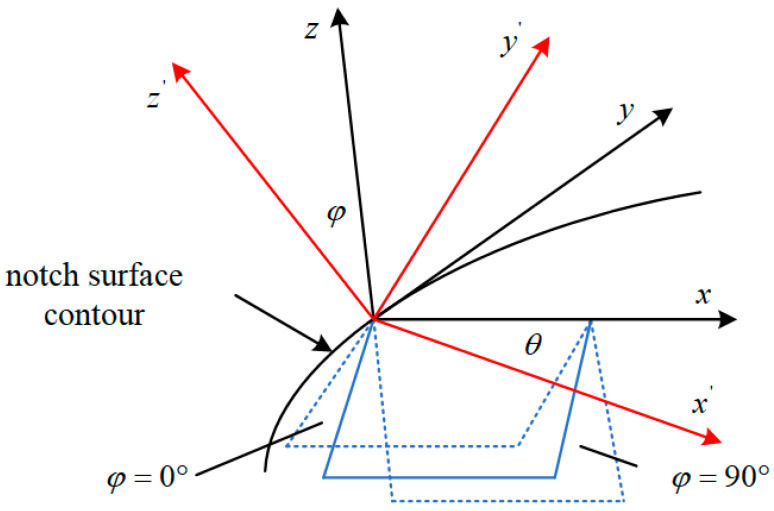
Coordinate transformation principle.

Determination of the strain and stress components at the dangerous point on the critical plane can be achieved through the coordinate transformation method, as illustrated in Figure 2.

The matrix *L* for coordinate transformation is shown below:

(2) $$L=\left[\begin{array}{ccc} \cos\theta & -\sin\theta \cos\varphi & \sin\theta \sin\varphi \\ \sin\theta & \cos\theta \cos\varphi & -\cos\theta \cos\varphi \\ 0 & \sin\varphi & \sin\varphi \end{array}\right]$$

In the new coordinate system, the transformed strain and stress components at critical points can be expressed as

(3) $$\sigma_{ij}^{\prime }=L\sigma_{ij}L^{T},\varepsilon_{ij}^{\prime }=L\varepsilon_{ij}L^{T}$$

where $L^{T}$ is the transpose matrix of *L*.

### 2.3. Determination of Energy-Critical Plane

First, the plastic strain energy density on planes with arbitrary orientations around the critical point is systematically evaluated by numerically adjusting the rotation angles θ and φ. This exhaustive numerical search ensures that all possible orientations are considered, allowing for the accurate identification of potential fatigue damage initiation sites. Among these planes, the one presenting the maximum plastic strain energy density is then identified and defined as the critical plane. This selection is based on the fundamental assumption that fatigue cracks tend to initiate and propagate along the plane where the highest localized plastic deformation and energy concentration occur. Finally, the critical plane is used to extract the relevant damage parameters, which are subsequently incorporated into multiaxial fatigue prediction models. In this way, the fatigue service life of metallic components under complex loading conditions can be estimated comprehensively and accurately. This approach not only improves prediction precision but also provides a more physically meaningful description of fatigue damage mechanisms, thereby enhancing its practical applicability and reliability in engineering applications.

Liu et al. [31] proposed the function of plastic strain energy density about and in an arbitrary plane.

(4) $$f\left(\theta ,\varphi \right)=\left(\sigma_{11}^{\prime }\varepsilon_{11}^{\prime }+\sigma_{12}^{\prime }\varepsilon_{12}^{\prime }+\sigma_{13}^{\prime }\varepsilon_{13}^{\prime }\right)/2$$

In Equation (4),

(5) $$\begin{array}{ll} \varepsilon_{11}^{\prime }= & \left(\varepsilon_{11}L_{11}^{T}+\varepsilon_{21}L_{12}^{T}+\varepsilon_{31}L_{13}^{T}\right)L_{11}+\left(\varepsilon_{12}L_{11}^{T}+\varepsilon_{22}L_{12}^{T}+\varepsilon_{32}L_{13}^{T}\right)L_{21}+\\ & \left(\varepsilon_{13}L_{11}^{T}+\varepsilon_{23}L_{12}^{T}+\varepsilon_{33}L_{13}^{T}\right)L_{31} \end{array}$$

(6) $$\begin{array}{ll} \varepsilon_{12}^{\prime }= & \left(\varepsilon_{11}L_{11}^{T}+\varepsilon_{21}L_{12}^{T}+\varepsilon_{31}L_{13}^{T}\right)L_{12}+\left(\varepsilon_{12}L_{11}^{T}+\varepsilon_{22}L_{12}^{T}+\varepsilon_{32}L_{13}^{T}\right)L_{22}+\\ & \left(\varepsilon_{13}L_{11}^{T}+\varepsilon_{23}L_{12}^{T}+\varepsilon_{33}L_{13}^{T}\right)L_{32} \end{array}$$

(7) $$\begin{array}{ll} \varepsilon_{13}^{\prime }= & \left(\varepsilon_{11}L_{11}^{T}+\varepsilon_{21}L_{12}^{T}+\varepsilon_{31}L_{13}^{T}\right)L_{13}+\left(\varepsilon_{12}L_{11}^{T}+\varepsilon_{22}L_{12}^{T}+\varepsilon_{32}L_{13}^{T}\right)L_{23}+\\ & \left(\varepsilon_{13}L_{11}^{T}+\varepsilon_{23}L_{12}^{T}+\varepsilon_{33}L_{13}^{T}\right)L_{33} \end{array}$$

(8) $$\begin{array}{ll} \sigma_{11}^{\prime }= & \left(\sigma_{11}L_{11}^{T}+\sigma_{21}L_{12}^{T}+\sigma_{31}L_{13}^{T}\right)L_{11}+\left(\sigma_{12}L_{11}^{T}+\sigma_{22}L_{12}^{T}+\sigma_{32}L_{13}^{T}\right)L_{21}+\\ & \left(\sigma_{13}L_{11}^{T}+\sigma_{23}L_{12}^{T}+\sigma_{33}L_{13}^{T}\right)L_{31} \end{array}$$

(9) $$\begin{array}{ll} \sigma_{12}^{\prime }= & \left(\sigma_{11}L_{11}^{T}+\sigma_{21}L_{12}^{T}+\sigma_{31}L_{13}^{T}\right)L_{12}+\left(\sigma_{12}L_{11}^{T}+\sigma_{22}L_{12}^{T}+\sigma_{32}L_{13}^{T}\right)L_{22}+\\ & \left(\sigma_{13}L_{11}^{T}+\sigma_{23}L_{12}^{T}+\sigma_{33}L_{13}^{T}\right)L_{32} \end{array}$$

(10) $$\begin{array}{ll} \sigma_{13}^{\prime }= & \left(\sigma_{11}L_{11}^{T}+\sigma_{21}L_{12}^{T}+\sigma_{31}L_{13}^{T}\right)L_{13}+\left(\sigma_{12}L_{11}^{T}+\sigma_{22}L_{12}^{T}+\sigma_{32}L_{13}^{T}\right)L_{23}+\\ & \left(\sigma_{13}L_{11}^{T}+\sigma_{23}L_{12}^{T}+\sigma_{33}L_{13}^{T}\right)L_{33} \end{array}$$

Let

(11) $${\left(⊗\right)}_{u}=\left[{\left(⊗\right)}_{11}-{\left(⊗\right)}_{22}\right]/2,{\left(⊗\right)}_{v}=\left[{\left(⊗\right)}_{11}+{\left(⊗\right)}_{22}\right]/2$$

where ⊗ represents either stress components or strain components.

In this condition, the formulation can be expressed as

(12) $$\begin{array}{ll} f\left(\theta ,\varphi \right)= & \frac{1}{2}\left[\sigma_{v}\varepsilon_{v}+\sigma_{u}\varepsilon_{u}+\sigma_{12}\varepsilon_{12}-\sigma_{33}\varepsilon_{33}-\left(\sigma_{v}\varepsilon_{u}+\sigma_{u}\varepsilon_{v}\right)\cos\left(2\theta \right)\right]\sin^{2}\varphi -\\ & \frac{1}{2}\left[\left(\sigma_{v}\varepsilon_{12}+\sigma_{12}\varepsilon_{v}\right)\sin\left(2\theta \right)\right]\sin^{2}\varphi +\sigma_{33}\varepsilon_{33} \end{array}$$

Since the function $f_{\left(\theta ,\varphi \right)}$ directly relates to plastic strain energy density, a higher energy density on a specific material plane indicates a greater likelihood of crack initiation and propagation in that direction. Identifying the stationary points of this function, which correspond to the maximum values of plastic strain energy density, allows for effective determination of the plane most vulnerable to fatigue crack initiation. Solving Equation (12) for the stationary points explicitly yields the critical angles and orientations that define the plane with the highest energy concentration, thereby enabling accurate prediction of where fatigue cracks are most likely to develop under cyclic loading.

(1) Partial derivative with respect to θ:

The partial derivative of Equation (12) in terms of θ is obtained as follows:

(13) $$\frac{\partial f(\theta ,\varphi )}{\partial \theta }=\left[\left(\sigma_{v}\varepsilon_{u}+\sigma_{u}\varepsilon_{v}\right)\sin\left(2\theta \right)-\left(\sigma_{v}\varepsilon_{12}+\sigma_{12}\varepsilon_{v}\right)\cos\left(2\theta \right)\right]\sin^{2}\varphi$$

Let $\frac{\partial f\left(\theta ,\varphi \right)}{\partial \theta }=0$, and the solution can be classified into the following two conditions:

Case (1): $\varphi =n\pi ,n=\left(0,1,2\right)$. This case is discarded as it does not conform to the physical reality.

Case (2): $\varphi \neq n\pi ,\tan(2\theta )=\left(\sigma_{v}\varepsilon_{12}+\sigma_{12}\varepsilon_{v}\right)/\left(\sigma_{v}\varepsilon_{u}+\sigma_{u}\varepsilon_{v}\right)$. This case conforms to the physical reality and is retained.

(2) Partial derivative with respect to φ:

The partial derivative of Equation (12) in terms of φ is obtained as follows:

(14) $$\begin{array}{ll} \frac{\partial f\left(\theta ,\varphi \right)}{\partial \varphi }= & \frac{1}{2}\left[\sigma_{v}\varepsilon_{v}+\sigma_{u}\varepsilon_{u}+\sigma_{12}\varepsilon_{12}-\sigma_{33}\varepsilon_{33}-\left(\sigma_{v}\varepsilon_{u}+\sigma_{u}\varepsilon_{v}\right)\cos\left(2\theta \right)\right]\sin\left(2\varphi \right)-\\ & \frac{1}{2}\left[\left(\sigma_{v}\varepsilon_{12}+\sigma_{12}\varepsilon_{v}\right)\sin\left(2\theta \right)\right]\sin\left(2\varphi \right) \end{array}$$

Let $\frac{\partial f\left(\theta ,\varphi \right)}{\partial \varphi }=0$, and we obtain $\varphi =90^{{}^{\circ}}\;or\;\varphi =0^{\circ }$.

Therefore, the position of the energy-critical plane can be identified as

(15) $$\theta =\frac{1}{2}\arctan\left(\frac{\sigma_{v}\varepsilon_{12}+\sigma_{12}\varepsilon_{v}}{\sigma_{v}\varepsilon_{u}+\sigma_{u}\varepsilon_{v}}\right),\;\varphi =90^{{}^{\circ}}$$

Equation (15) shows that its position can be uniquely identified once the stress components or strain components at the critical points is given.

## 3. Damage Parameters Analysis of Multiaxial Fatigue Model

### 3.1. Existing Multiaxial Fatigue Model

#### 3.1.1. SWT Model

Smith, Watson, and Topper [32] proposed that fatigue behavior is primarily governed by the combined effect of the maximum normal stress and the strain amplitude experienced by a material during cyclic loading. Based on this observation, they introduced the concept of a critical plan. On this plane, the corresponding maximum normal stress is used together with the strain amplitude to evaluate fatigue damage. The SWT model is expressed as follows:

(16) $$\sigma_{n,\max}\varepsilon_{a}=\frac{{\left(\sigma_{f}^{\prime }\right)}^{2}}{E}{\left(2N_{f}\right)}^{2b}+\sigma_{f}^{\prime }\varepsilon_{f}^{\prime }{\left(2N_{f}\right)}^{b+c}$$

where $\sigma_{n,\max}$ and $\varepsilon_{a}$ are the maximum normal stress and the normal strain amplitude; $\sigma_{f}^{\prime }$, *b*, $\varepsilon_{f}^{\prime }$, and *c* are the fatigue strength coefficient, fatigue strength exponent, fatigue ductility coefficient and fatigue ductility exponent, respectively; $N_{f}$ is the fatigue life; *E* is the Young’s modulus.

#### 3.1.2. Equivalent Strain Model

In engineering practice, the equivalent strain approach is frequently employed for estimating the lifespan of structures or components under uniaxial tension or predominantly uniaxial loading conditions [33]. This method relates the macroscopic mechanical properties of a material, including its elastic modulus, yield strength, and fatigue strength, to its fatigue performance under cyclic loading. By reducing complex cyclic loading histories to an equivalent strain parameter, this approach enables practical and relatively accurate fatigue life predictions, especially in situations where multiaxial stress effects are negligible. This class of models is generally referred to as strain-based fatigue models, and is typically represented by the following mathematical expression:

(17) $$\frac{\Delta \varepsilon_{eq}}{2}=\frac{\sigma_{f}^{\prime }}{E}{\left(2N_{f}\right)}^{b}+\varepsilon_{f}^{\prime }{\left(2N_{f}\right)}^{c}$$

where $\Delta \varepsilon_{eq}$ is equivalent strain amplitude.

#### 3.1.3. FS Model

Fatemi and Socie [16] found that under multiaxial loading conditions, fatigue behavior should be evaluated. Based on extensive experimental investigations and theoretical considerations, they identified the critical plane as the one where the maximum shear strain amplitude occurs during cyclic loading. To capture this interaction, they proposed that the fatigue parameter should not only consider the shear strain amplitude but also account for the detrimental effect of tensile normal stress on the critical plane. The model is typically expressed as follows:

(18) $$\frac{\Delta \gamma_{\max}}{2}\left(1+k\frac{\sigma_{n,\max}}{\sigma_{y}}\right)=\frac{\tau_{f}^{\prime }}{G}{\left(2N_{f}\right)}^{b_{0}}+\gamma_{f}^{\prime }{\left(2N_{f}\right)}^{c_{0}}$$

where $\Delta \gamma_{\max}/2$ is the maximum shear strain amplitude; $\sigma_{y}$ is the yield stress of the material; *G* is the shear modules; $b_{0}$ and $c_{0}$ are the shear fatigue strength exponent and shear fatigue ductility exponent, respectively; $\tau_{f}^{\prime }$ and $\gamma_{f}^{\prime }$ are the shear fatigue strength coefficient and shear fatigue ductility coefficient; k is a material constant.

### 3.2. Proposed Multiaxial Fatigue Life Prediction Model

Under complex loading, the principal stress and strain axes in metallic materials continuously rotate, causing changes in slip system orientations and destabilizing the dislocation structure [34]. This dynamic stress evolution alters the activation sequence of slip systems, making deformation more complex than under proportional or uniaxial loading. In out-of-phase tension-torsion loading, the rotation of the principal axes allows more grains to reach orientations favorable for slip. This effect is especially significant in materials with heterogeneous microstructures and diverse crystallographic textures, where grains respond differently to the changing stress state.

**Figure 3:**
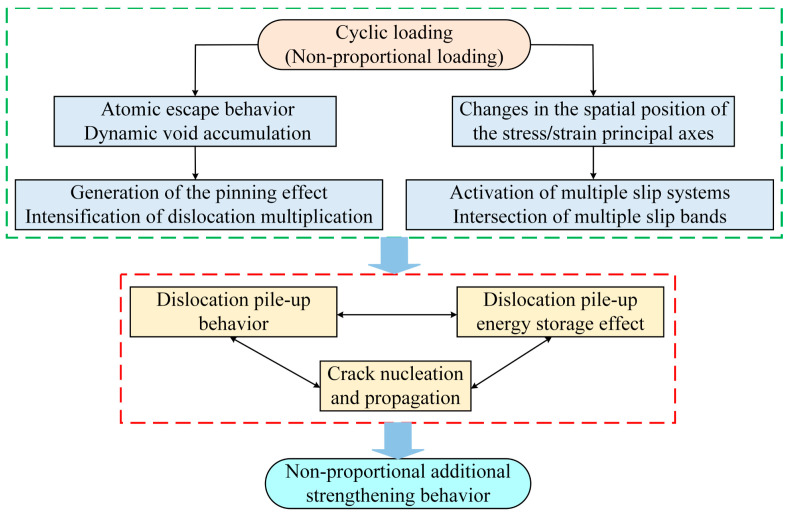
Mechanism of this additional strengthening effect.

As a result, interactions among multiple activated slip systems become stronger, leading to increased dislocation accumulation and pile-up at grain boundaries or other microstructural barriers. These localized concentrations of stress and strain promote the initiation of microcracks, even at relatively low applied stress levels. This process is widely recognized as the main mechanism behind the additional damage observed under non-proportional multiaxial loading. Such mechanisms often reduce the fatigue resistance of metallic components and should be considered in the design of structures subjected to complex service loading. Figure 3 provides a schematic illustration of this additional strengthening mechanism.

#### 3.2.1. Numerical Estimation of the Additional Strengthening Coefficient

Kanazawa et al. [35] quantitatively characterized the additional strengthening effect of the material by defining a non-proportional coefficient, as shown in Equation (19).

(19) $$\alpha =\frac{\sigma_{NP}}{\sigma_{IP}}-1$$

where $\sigma_{NP}$ represents the equivalent stress corresponding to $90^{\circ }$ circular loading path during the stabilized plastic deformation stage, whereas $\sigma_{IP}$ represents the equivalent stress under in-phase loading.

When Equation (19) is applied to evaluate a material’s additional strengthening coefficient, it requires stress parameters obtained under stable plastic strain conditions. However, in practical scenarios, particularly under complex non-proportional loading, plastic deformation often takes a considerable amount of time to reach a stable state. This requirement significantly complicates the process of experimental data acquisition, as it involves extensive and tightly controlled testing procedures. As a result, direct determination of the additional strengthening coefficient using this method becomes impractical in many engineering applications. The present study addresses this limitation by proposing an alternative approach in which the additional strengthening coefficient α and the static strengthening coefficient β are fitted using experimental data derived from non-proportional loading conditions. This method establishes empirical relationships between the two coefficients and readily measurable material properties. As a result, it enables numerical estimation of the additional strengthening coefficient without the need for fully stabilized plastic strain conditions. The outcome is a significant improvement in computational efficiency and an expansion in the applicability of the method.

**Figure 4:**
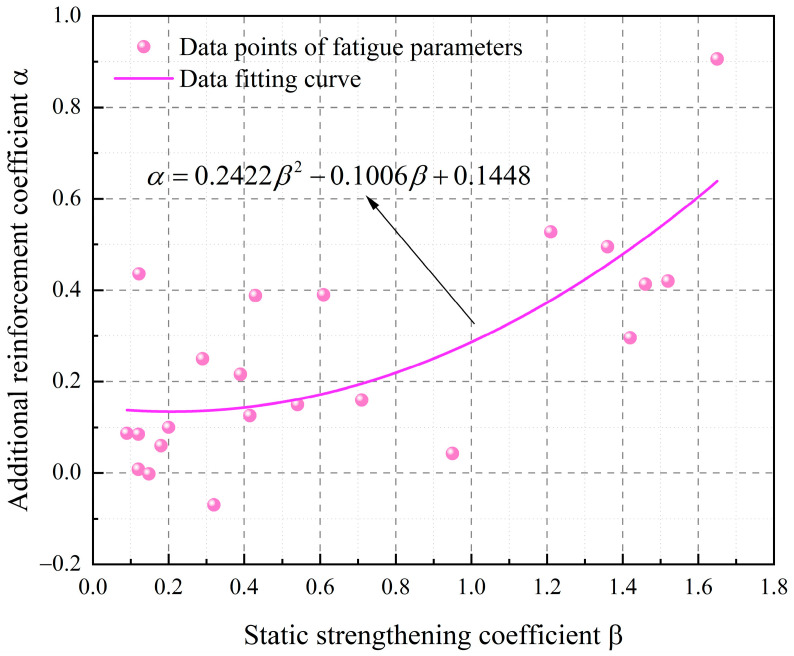
Fitting results of the additional strengthening coefficient for various materials.

This fitting-based approach was validated for its effectiveness and reliability using data from 22 different metallic materials compiled from the literature [36]. These data were presented in Table 1. These materials constitute a comprehensive dataset appropriate for regression analysis. Figure 4 demonstrates the results of the fitting process and reveals a strong correlation between the additional strengthening coefficient and key material parameters. This outcome provides a predictive framework for estimating strengthening behavior across various materials.

The accuracy and stability of the fitting procedure were improved by selecting a mathematical function with superior performance and adaptability. Compared to conventional regression methods, the chosen function more effectively captures the nonlinear relationships between the influencing variables and the additional strengthening effect. Using this enhanced approach, an approximate formula for estimating the additional strengthening coefficient was derived. This formula not only simplifies the estimation process but also demonstrates strong consistency with experimental observations, thereby enhancing both its practical applicability and theoretical validity.

(20) $$\alpha =0.2422\beta^{2}-0.1006\beta +0.1448$$

(21) $$\beta =\frac{\sigma_{b}}{\sigma_{y}}-1$$

where $\sigma_{b}$ is the tensile strength.

Using the aforementioned method, the additional strengthening coefficients for the three metallic materials were obtained, as presented in Table 2.

#### 3.2.2. Proposed Model Construction

Under the same equivalent strain conditions, specimens subjected to non-proportional loading exhibit significantly shorter fatigue lives compared with those under proportional loading. This reduction is primarily attributed to the disruption of the dislocation structure caused by the continuous rotation of principal stress and strain axes [40]. Such reorientation increases the frequency of interactions between dislocations and microstructural obstacles, such as grain boundaries and precipitates, resulting in accelerated damage accumulation and premature fatigue failure.

Most existing prediction models consider strain as the dominant damage parameter, while often overlooking the critical influence of normal stress. Under multiaxial loading conditions, however, normal stress directly affects fatigue crack growth rates. As a result, relying solely on strain-based criteria fails to reflect the complex interactions among stress state, material hardening, and crack growth mechanisms, particularly under out-of-phase loading paths. This limitation is addressed in the present study through the introduction of a correction factor μ that captures the interaction between the maximum normal stress and the additional strengthening coefficient. By incorporating both mechanical loading conditions and material-specific hardening responses, this factor enables a more comprehensive representation of the fatigue damage mechanism, which is defined as

(22) $$\mu =1+\frac{\sigma_{n,\max}}{\sigma_{y}}\left(1+\alpha \right)$$

Under multiaxial loading, materials exhibit a clear trend of fatigue life reduction as the phase difference between stress components increases [41]. A larger phase difference intensifies the effects of non-proportional loading, accelerates fatigue damage, and results in shorter fatigue life. In particular, when the phase difference φ reaches $90^{\circ }$, the fatigue life is minimized, indicating that the non-proportional strengthening effect reaches its peak. This observation suggests that the destructive interaction between different stress components becomes most pronounced at this phase angle. Improved accuracy in fatigue life prediction under asynchronous or out-of-phase multiaxial loads requires the inclusion of a phase difference correction factor λ, which is defined as

(23) $$\lambda =1+\frac{\sin^{2}\varphi }{2}$$

This study establishes a comprehensive parameter, referred to as the non-proportional additional damage coefficient $f_{np}$, by systematically integrating two previously defined influence factors. The coefficient $f_{np}$ quantitatively characterizes the cumulative effect of the additional strengthening behavior induced by complex multiaxial non-proportional loading. Specifically, it captures both stress-driven material hardening and loading-path non-proportionality. Through this integration, the proposed model achieves a more complete and physically meaningful representation of the fatigue damage mechanism. As a result, the model more accurately reflects real-world fatigue behavior and demonstrates significant improvements in both the accuracy and reliability of predictions. The mathematical expression for the non-proportional additional damage coefficient $f_{np}$ is given as Equation (24):

(24) $$f_{np}=\sqrt{\mu \lambda }=\sqrt{\left[1+\frac{\sigma_{n,\max}}{\sigma_{y}}\left(1+\alpha \right)\right]\left(1+\frac{\sin^{2}\varphi }{2}\right)}$$

**Figure 5:**
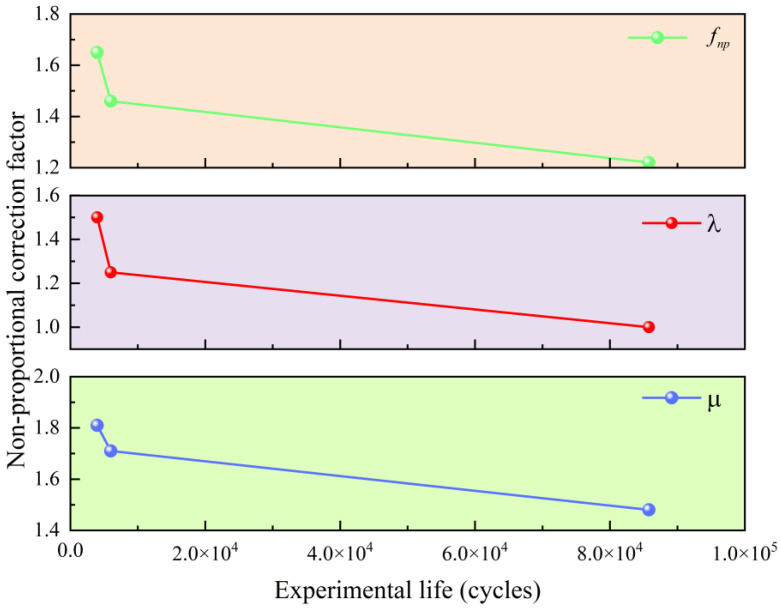
The relationship between the correction factors and the experimental life.

As shown in Figure 5, the relationship between the three factors, namely μ, λ, and $f_{np}$, and the experimental life is clearly demonstrated.

It can be observed that as the experimental fatigue life decreases, all three proposed factors exhibit a consistent downward trend, although at different rates. In particular, shorter fatigue lives correspond to higher values of these correction parameters, indicating a strong inverse relationship between experimental life and the three influencing factors. These trends align well with established fatigue theories and empirical evidence: shorter fatigue lives are generally associated with more pronounced additional strengthening effects and greater accumulation of non-proportional damage. Moreover, the close agreement between predicted values and experimental data strongly supports the assumptions underlying the proposed fatigue life model. This consistency confirms both the theoretical validity of the model and the practical significance of incorporating these correction factors to enhance prediction accuracy under complex multiaxial loading conditions

While the equivalent strain model performs adequately under proportional loading, its predictive accuracy deteriorates under non-proportional conditions. As discussed in the previous section, this limitation arises primarily because the model does not account for several critical mechanisms, including additional strengthening effects induced by complex loading paths, phase differences, and variations in maximum normal stress. These omissions lead to significant discrepancies between predicted and actual fatigue lives under realistic multiaxial, non-proportional loading scenarios. To address these limitations, this study proposes a correction strategy with two key components. The first component introduces a correction factor μ that reflects the combined influence of maximum normal stress and the intrinsic material strengthening characteristics α, thereby improving the representation of fatigue resistance under complex loading. The second component λ involves a phase-difference-related correction factor, which enhances prediction accuracy under asynchronous multiaxial loading by capturing the effects of stress–strain path deviations on internal strengthening behavior.

Incorporating these correction factors into the fatigue life model increases both its sensitivity and predictive accuracy, particularly in scenarios where conventional models fail. The improved model presented in this study provides a practical analytical framework for engineering fatigue assessment, effectively capturing the complex interactions among stress and strain variables, critical plane orientation, and material-specific strengthening behaviors.

(25) $$\sqrt{{\left(\varepsilon_{n}\right)}^{2}+\frac{{\left(\Delta \gamma_{\max}\right)}^{2}/2}{3}}\sqrt{\left[1+\frac{\sigma_{n,\max}}{\sigma_{y}}\left(1+\alpha \right)\right]\left(1+\frac{\sin^{2}\varphi }{2}\right)}=\frac{\sigma_{f}^{\prime }}{E}{\left(2N_{f}\right)}^{b}+\varepsilon_{f}^{\prime }{\left(2N_{f}\right)}^{c}$$

where $\varepsilon_{n}$ is normal strain.

## 4. Finite Element Analysis of Metallic Materials

### 4.1. Material and Geometric Parameters

A comprehensive evaluation of the applicability of the proposed model under various loading conditions was conducted through detailed finite element simulations based on experimental data from three widely used engineering materials: En8 [37] (a medium-carbon steel), TC4 [38] (a titanium alloy), and Al7050-T7451 [39] (a high-strength aluminum alloy). These materials were selected to represent different categories and mechanical properties, thereby allowing validation of the proposed model across a broad range of microstructural characteristics and demonstrating its general applicability and robustness.

**Figure 6:**
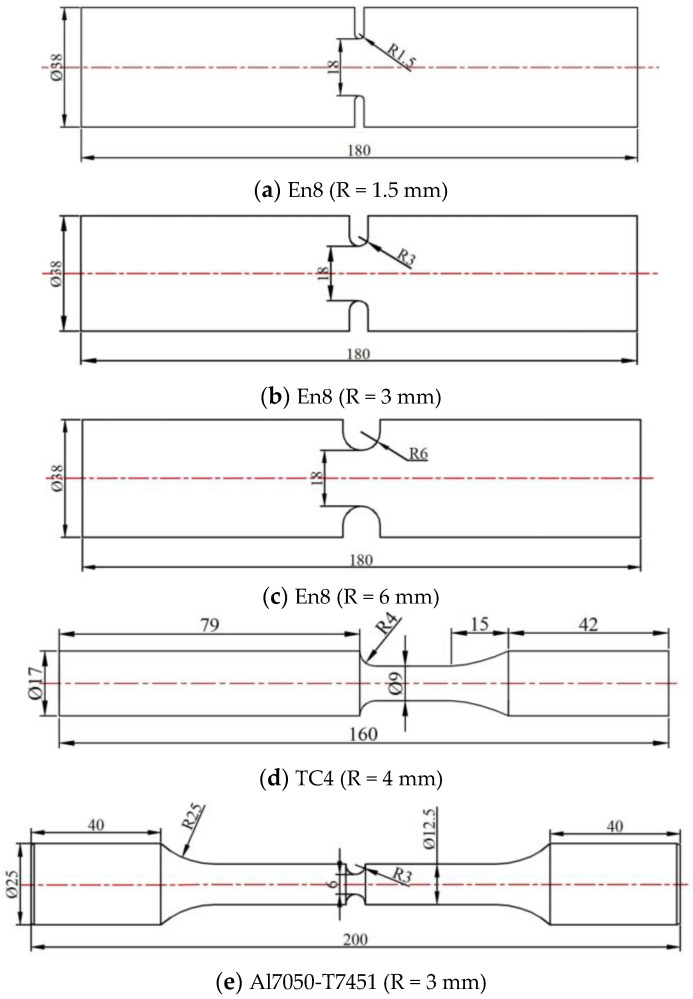
Notched specimens.

Their mechanical and fatigue performance parameters are summarized in Table 3, while the corresponding fatigue test results are presented in Table 4, Table 5 and Table 6. Figure 6 illustrates the geometrical configuration and dimensions of the notched specimens used in the simulation.

### 4.2. Stress and Strain Analysis

**Figure 7:**
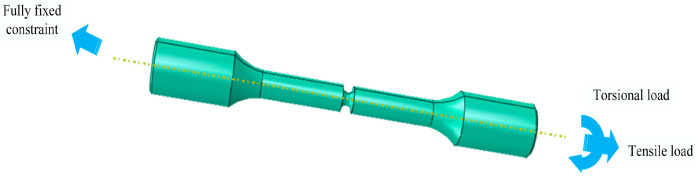
Boundary conditions.

The finite element method is widely applied in fatigue durability evaluation and life estimation of engineering components [42]. In this study, finite element analysis was conducted using Abaqus to capture detailed stress and strain distributions at the notch roots under different loads. These simulation results provide essential input data for identifying the critical plane, which plays a key role in multiaxial fatigue analysis. The boundary conditions were defined such that one end of the notched specimen was fully constrained, while the other end experienced combined tension and torsion loading to simulate actual service conditions. The boundary conditions of the model are shown in Figure 7.

**Figure 8:**
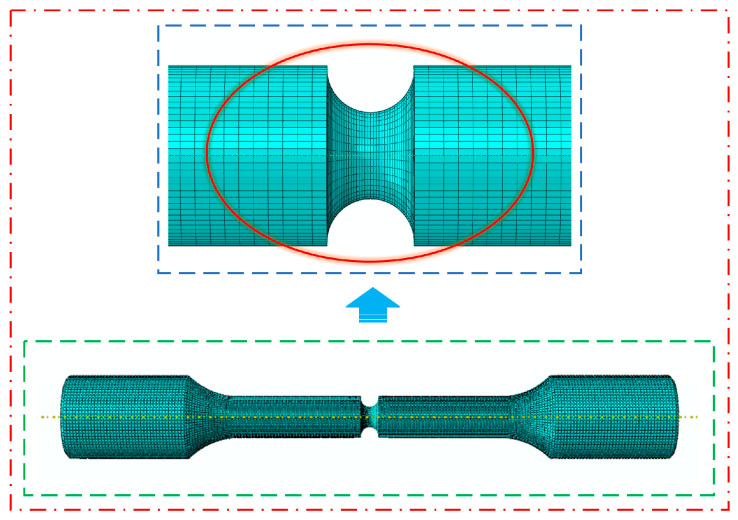
Mesh layout of the notched Al7050-T7451 specimen.

Accuracy and efficiency were achieved by using a structured hexahedral mesh, which provides better numerical stability and faster convergence than tetrahedral elements. Particular attention was paid to mesh refinement near the notch, where stress concentrations are highest. For example, in the Al7050-T7451 specimen, the mesh size was reduced to 0.6 mm in the high-stress region near the notch, while the rest of the specimen was meshed uniformly at 1 mm. This approach strikes a balance between computational cost and solution accuracy. Figure 8 shows the mesh layout of the notched Al7050-T7451 specimen, highlighting the refined region around the notch.

**Figure 9:**
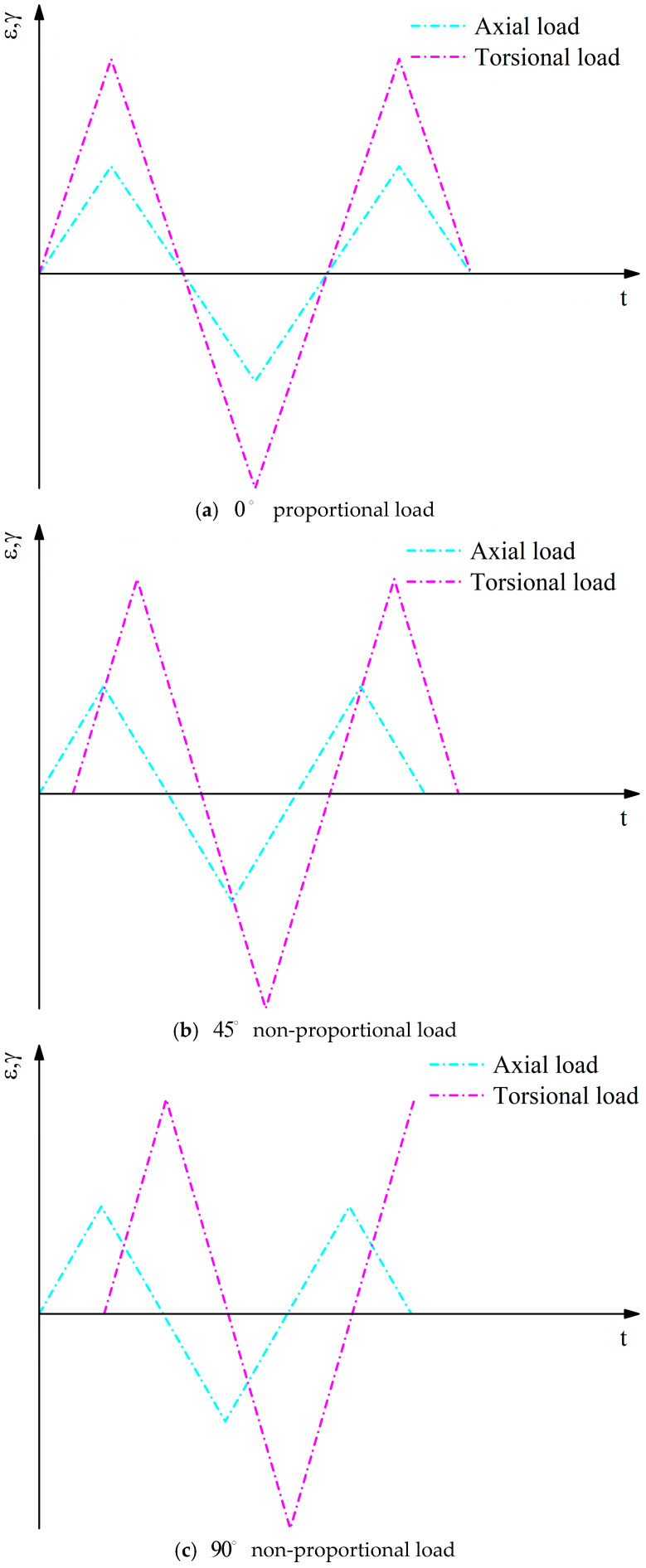
Loading waveforms.

When performing the finite element analysis, different load histories and loading waveforms are applied to achieve tensile and torsional loads with various phase differences. Since all five notched specimens in the multiaxial fatigue experiments were tested under constant amplitude loading, the load history was set as a triangular waveform. Simulations were conducted under 0° proportional loading, 45° non-proportional loading, and 90° non-proportional loading conditions. The specific loading waveforms are shown in Figure 9.

**Figure 10:**
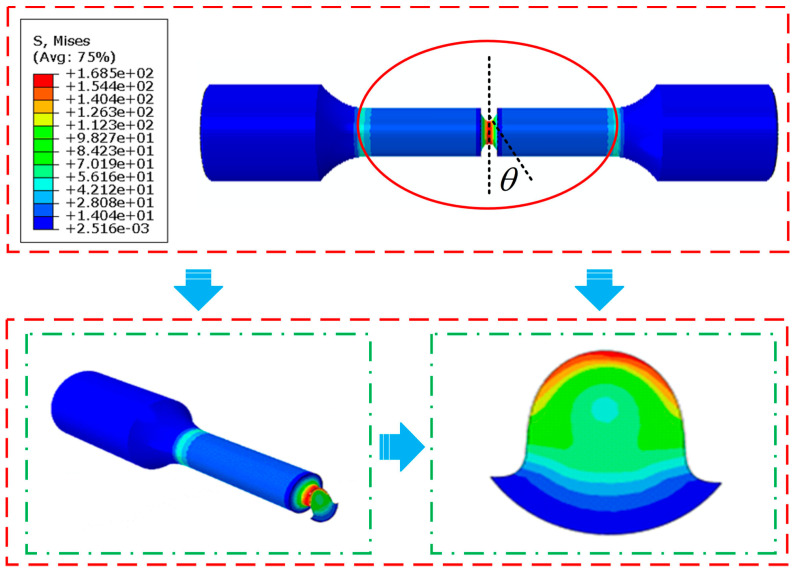
Al7050-T7451 equivalent stress cloud diagram of notched specimen.

The plane with the maximum strain energy for the Al7050-T7451 specimen under the first loading condition was identified by analyzing the equivalent stress distribution on candidate planes. Using the finite element simulation results at the notch and applying Equations (4)–(15), the critical angle θ was determined. The specimen was rotated by $\theta =23^{\circ }$ in Abaqus so that the working plane coincided with the critical plane. A sectional cut was made along the working plane at $\theta =23^{\circ }$, and the resulting equivalent stress distribution on the critical plane is shown in Figure 10.

## 5. Model Validation and Comparison

### 5.1. Fatigue Life Prediction

The proposed model was experimentally validated using three different materials: En8, TC4, and Al7050-T7451. Its performance was further assessed through a comparative analysis involving three conventional fatigue life prediction models.

**Figure 11:**
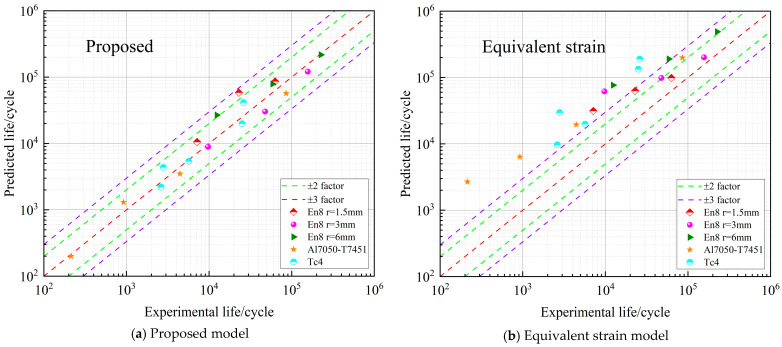

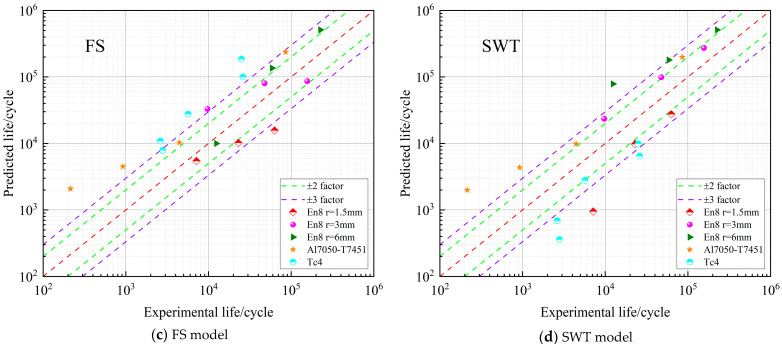
Life dispersion zone diagram under proportional loading.

Figure 11 illustrates the comparative assessment between the experimentally obtained results and the predicted values corresponding to proportional loading conditions.

As depicted in Figure 11, the proposed model (Figure 11a) exhibits excellent predictive performance for proportional loading. Most of the calculated results fall inside the double-dispersion zone, indicating that the model captures the fatigue behavior under simple loading conditions with high accuracy. By contrast, the conventional models—including the Equivalent Strain model (Figure 11b), the FS model (Figure 11c), and the SWT model (Figure 11d)—show much poorer agreement with the experimental results. A considerable portion of their predictions lies outside the double-dispersion zone, and only a few points reach the triple-dispersion zone. These observations clearly demonstrate their limited predictive capability for proportional loading conditions.

**Figure 12:**
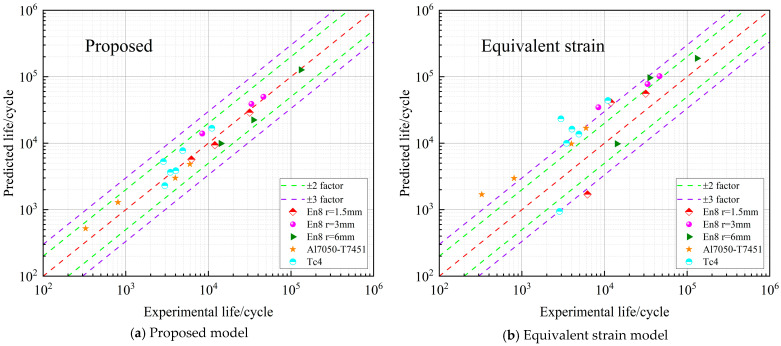

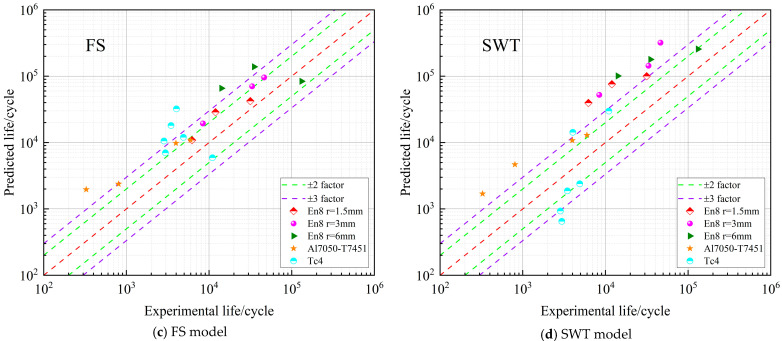
Life dispersion zone diagram under non-proportional loading.

Figure 12 provides a comparison between the experimentally measured results and the corresponding model predictions under non-proportional loading conditions.

Figure 12 presents the results obtained under non-proportional loading, in which the loading paths are more complex. As shown in Figure 12a, the proposed model still achieves excellent prediction accuracy, with all predicted fatigue lives located inside the double-dispersion zone. This indicates that the model is capable of accounting for the additional strengthening effects caused by multiaxial loading. By contrast, the other models (Figure 12b–d) show poor performance, as most of their predicted values fall outside even the triple-dispersion zone, again revealing their limited capability under non-proportional loading.

### 5.2. Error Analysis and Discussion 

A probabilistic error analysis was conducted to further quantify and compare the performance of the models. The prediction error, denoted as $P_{error}$, is defined as

(26) $$P_{error}=\log_{10}\left(\frac{N_{P}}{N_{e}}\right)$$

where $N_{P}$ is the estimated lifespan, $N_{e}$ is the tested lifespan.

**Figure 13:**
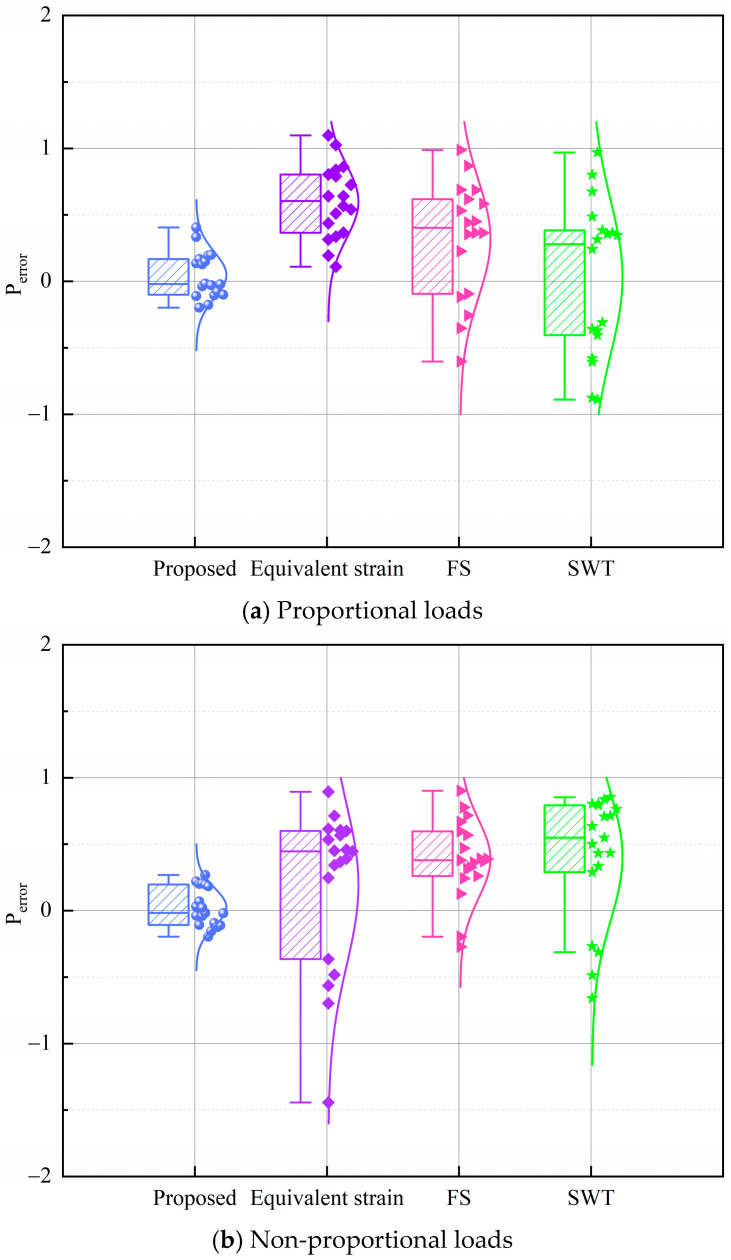
Box plots for different models.

Box plots combined with fitted normal distribution curves were employed to systematically characterize the distribution of prediction errors for each model. In this representation, a positive $P_{error}$ error denotes overestimation, while a negative $P_{error}$ error indicates underestimation. The width of each box corresponds to the standard deviation, and the superimposed curve represents the fitted normal distribution. Collectively, these graphical elements offer a clear depiction of both the concentration and dispersion of prediction errors. A critical metric of model performance is the proximity of the mean prediction error to zero. When the mean error approaches zero and the standard deviation is small, the resulting normal distribution curve exhibits a tall and narrow shape, indicative of high predictive accuracy and low variability. Such performance is particularly significant under non-proportional loading conditions, where accurate fatigue life predictions are inherently more challenging. Figure 13 presents the box plots and normal distribution curves of prediction errors for the evaluated models.

As illustrated in Figure 13, the proposed model consistently demonstrates a mean prediction error approaching zero across diverse loading conditions, while exhibiting relatively small standard deviations. Consequently, the corresponding normal distribution curves are tall and narrow, indicative of high predictive accuracy, low variability, and superior precision. The close alignment of predicted fatigue lives with experimental values further attests to the model’s stability and reliability. In contrast, the three conventional models display mean errors that deviate considerably from zero, accompanied by substantially larger standard deviations. Their normal distribution curves are thus shorter and broader, reflecting increased dispersion, reduced precision, and greater uncertainty in fatigue life predictions. These observations highlight the limited consistency and predictive capability of conventional models under complex and variable loading conditions.

Collectively, this comparative analysis unequivocally demonstrates that the proposed model delivers accurate and reliable predictions across all three notched materials and a variety of loading scenarios. Its robust adaptability to different stress states and complex loading patterns underscores its potential for practical engineering applications in fatigue assessment. The findings substantiate the predictive advantages and general applicability of the proposed model, providing valuable insights for the advancement of fatigue life estimation methodologies.

## 6. Conclusions

This study presents a novel fatigue life estimation model specifically developed for notched metallic materials, emphasizing the critical role of additional strengthening effects. The principal findings and conclusions are summarized as follows:

(1) A novel methodology has been proposed to estimate the additional strengthening coefficient based on the static strengthening coefficient. This approach provides a practical and efficient means to account for material hardening in multiaxial fatigue analyses. Building upon this foundation, a fatigue life prediction model was formulated through the integration of an energy-based framework with the critical plane theory.

(2) Experimental validation demonstrates that the proposed model consistently achieves high predictive accuracy. The majority of predicted fatigue lives reside within the double-dispersion zone, with only minor deviations observed. In contrast, conventional models—including the Equivalent Strain, FS, and SWT models—exhibit markedly lower accuracy, with numerous predictions falling outside acceptable limits and exhibiting considerable scatter. These observations underscore the limitations of traditional models under complex multiaxial loading conditions.

(3) Statistical analysis further substantiates the strong robustness of the proposed model, which exhibits the lowest dispersion in prediction errors among all evaluated models. Such reduced variability enhances both the consistency and reliability of predictions across diverse loading conditions. These findings highlight the necessity of explicitly incorporating non-proportional strengthening effects into fatigue life prediction models.

## Figures and Tables

**Table 1:** Performance parameters of materials [36].

No.	Materials	$\sigma_{y}$ (MPa)	$\sigma_{b}$ (MPa)	β	α
1	AA6061	320	350	0.09	0.087
2	BT-9	865	970	0.12	0.085
3	VT-9	865	973	0.12	0.008
4	42CrMo4	980	1100	0.122	0.436
3	BT-10	485	557	0.148	−0.002036
6	2CrNiMoV	600	710	0.18	0.06
7	In718	1172	1407	0.2	0.1
8	S460N	500	643	0.29	0.25
9	OFHC(CU)	182	240	0.32	−0.0698
10	S25C	354	493	0.39	0.21558
11	S45C	445	630	0.415	0.125786
12	S55C	484	695	0.43	0.388372
13	6061A1	253	390	0.54	0.1494
14	CK45	410	660	0.609	0.39
15	SGV410	275	470	0.71	0.1597
16	1Cr-18	310	605	0.95	0.04285
17	SUS316	260	575	1.21	0.5277
18	SS347	250	590	1.36	0.495
19	SUS310S	215	520	1.42	0.2958
20	SS316L	230	565	1.46	0.413
21	800H	200	530	1.52	0.42
22	SS304	260	690	1.65	0.906

**Table 2:** Mechanical performance parameters of materials.

No.	Materials	$\sigma_{y}$ (MPa)	$\sigma_{b}$ (MPa)	β	α
1	En8 [37]	453	852.3	0.8815	0.2443
2	TC4 [38]	842.5	1045	0.2404	0.1346
3	Al7050-T7451 [39]	455	1054	1.3165	0.4321

**Table 3:** Performance parameters of materials.

Materials	E/GPa	$\sigma_{f}^{\prime }$/MPa	$\varepsilon_{f}^{\prime }$/MPa	*b*	*c*	$v_{e}$	$k^{\prime }$	$n^{\prime }$
En8	210	852.3	0.477	−0.105	−0.554	0.3	971.5	0.188
TC4	108.4	116.9	0.579	−0.049	−0.679	0.3	1054	0.0195
Al7050-T7451	70.3	731.98	0.6145	−0.8235	−0.7885	0.33	1096	0.0722

**Table 4:** Experimental results of En8 [37].

r/mm	No.	$\varphi^{\circ }$	$F_{a}(kN)$	$T_{a}(N.m)$	$N_{f}(Cycles)$
6	1	0	39.3	114.8	225,655
	2	0	49.8	126.3	58,662
	3	0	67.2	170.7	12,423
	4	90	49.6	114.2	131,784
	3	90	61.8	148.4	35,127
	6	90	69.5	186.5	14,146
3	7	0	40.4	102.72	156,422
	8	0	49	123	47,739
	9	0	60.6	178.8	9725
	10	90	46	140	46,428
	11	90	51	132.5	33,269
	12	90	63.6	181.7	8428
1.5	13	0	39.1	101.8	63,012
	14	0	44.1	120.5	22,974
	15	0	55.1	153.7	7156
	16	90	41.3	110.6	31,594
	17	90	45.1	125.6	11,989
	18	90	55.8	158.1	6229

**Table 5:** Experimental results of TC4 [38].

r/mm	No.	$\varphi^{\circ }$	$F_{a}(kN)$	$T_{a}(N.m)$	$N_{f}(Cycles)$
4	1	0	15	75	2627
	2	0	35	50	5690
	3	0	40	45	2793
	4	0	12	60	25,106
	5	0	20	50	26,106
	6	45	15	75	2969
	7	45	35	50	4037
	8	45	40	45	3470
	9	90	15	75	2855
	10	90	40	45	4902
	11	90	35	50	11,019

**Table 6:** Experimental results of Al7050-T7451 [39].

r/mm	No.	$\varphi^{\circ }$	$F_{a}(kN)$	$T_{a}(N.m)$	$N_{f}(Cycles)$
3	1	0	4.6133	3.8853	85,778
	2	0	11.4995	9.6061	214
	3	0	6.9227	5.9096	4447
	4	0	9.1983	7.8954	922
	3	45	6.9165	5.9055	5984
	6	90	6.8907	5.9276	3981
	7	90	11.4812	9.9487	327
	8	90	9.2128	7.9224	804

## Data Availability

The raw data supporting the conclusions of this article will be made available by the authors on request.

## Nomenclature

*b*	Fatigue strength exponent
*c*	Fatigue ductility exponent
*E*	Young modulus
*G*	Shear modulus
$b_{0}$	Shear fatigue strength exponent
$c_{0}$	Shear fatigue ductility exponent
K′	Cyclic strain hardening exponent
k	Material constant
n′	Unit complex root
$N_{f}$	Number of cycles to failure
$N_{P}$	Estimated lifespan
$N_{e}$	Tested lifespan
$\Delta \gamma_{\max}$	Maximum shear strain
$\gamma_{f}^{\prime }$	Shear fatigue ductility coefficient
$\tau_{f}^{\prime }$	Shear fatigue strength coefficient
$\varepsilon_{a}$	Normal strain amplitude
$\Delta \varepsilon_{eq}$	Equivalent strain amplitude
$\varepsilon_{f}^{\prime }$	Fatigue ductility coefficient
$\varepsilon_{n}$	Normal strain
$\sigma_{b}$	Tensile strength
$\sigma_{f}^{\prime }$	Fatigue strength coefficient
$\sigma_{IP}$	Equivalent stress under in-phase loading.
$\sigma_{NP}$	Equivalent stress corresponding to $90^{\circ }$ circular loading path
$\sigma_{n,\max}$	Maximum normal stress
$\sigma_{y}$	Yield strength
$v_{e}$	Poisson’s ratio
φ	Phase angle between tensional strain and torsional strain
